# 

**Published:** 1886-01

**Authors:** 


### BOOKS AND PAMPHLETS RECEIVED.

OUR LIVING WORLD, an artistic edition of the Rev. G. Wood's Natural History of Animated Creation. Published by Selmar Hess, New York.

THIRD BIENNIAL REPORT OF THE STATE BOARD OF HEALTH OF THE STATE OF IOWA. 1885.

STUDIES FROM THE BIOLOGICAL LABORATORY—JOHNS HOPKINS UNIVERSITY, BALTIMORE. Vol. III, No. 4. September, 1885.

BULLETIN OF THE ILLINOIS STATE LABORATORY OF NATURAL HISTORY. Index to Vol. I. Nos. 1, 2 and 3, Vol. II. Normal, Ill.

LECTURES ON THE SCIENCE OF AGRICULTURE. By Flavel S. Thomas, A.M., M.D. Hanson, Mass.

OBSERVATIONS UPON THE MUTUAL RELATIONS OF THE MEDICAL PROFESSION AND THE STATE. An Address delivered before the State Medical Society at Port Huron, Lansing, Mich.

AVENA SATIVA IN THE TREATMENT OF OPIUM ADDICTION. By J. B. Mattison, M.D. Brooklyn.

THE THERAPEUTICS OF HIGH TEMPERATURES IN YOUNG CHILDREN. By William Perry Watson, A.M., M.D., of Jersey City. Philadelphia: 1885.

FIRST ANNUAL REPORT NEW YORK CANCER HOSPITAL. 1885.

SIXTEENTH ANNUAL REPORT OF THE MANHATTAN EYE AND EAR HOSPITAL. New York: 1885.

DESCRIPTION OF THE HESPEROMYS TRUEI; A New Species belonging to the sub-family Murinæ. By R. W. Shufeldt, M.D., U. S. Army.

